# Dissecting the impact of N-acetylmannosamine (ManNAc) on ganglioside levels in a sialin-deficient cell model

**DOI:** 10.17912/micropub.biology.001733

**Published:** 2025-09-02

**Authors:** Marya S. Sabir, Marjan Huizing, William A. Gahl, Frances M. Platt, May Christine V. Malicdan

**Affiliations:** 1 National Human Genome Research Institute, National Institutes of Health, Bethesda, MD, USA; 2 NIH Oxford-Cambridge Scholars Program, University of Oxford, Oxford, UK; 3 Department of Pharmacology, University of Oxford, Oxford, UK

## Abstract

Lysosomal free sialic acid storage disorder (FSASD) is an ultra-rare neurodegenerative condition caused by mutations in *SLC17A5* , which encodes the lysosomal sialic acid exporter, sialin. Deficiency of sialin leads to lysosomal accumulation of unconjugated (“free”) sialic acid. This study investigated the ability of N-acetylmannosamine (ManNAc), a precursor of sialic acid, to rescue glycosphingolipid (GSL) sialylation in a SLC17A5-deficient HEK-293T model system. Our findings reveal that while ManNAc supplementation may enhance sialic acid biosynthesis, it does not fully restore ganglioside sialylation to wild-type levels, highlighting the essential role of lysosomal sialic acid recycling in maintaining GSL sialylation homeostasis.

**Figure 1. Effects of N-acetylmannosamine (ManNAc) supplementation on lysosomal volume and glycosphingolipid (GSL) profiles in SLC17A5-deficient HEK-293T cells:**
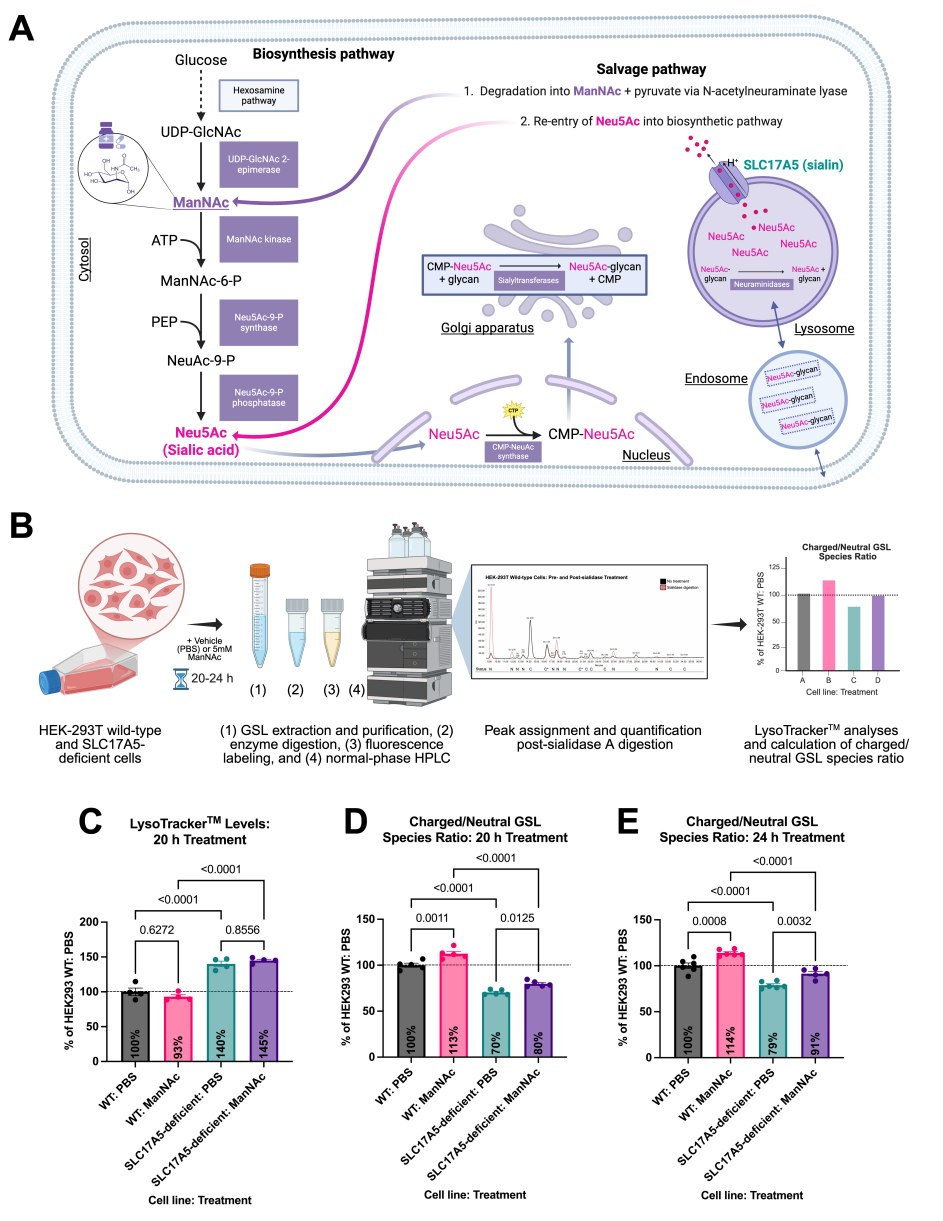
**(A)** Schematic overview of sialic acid biosynthesis and salvage pathways, highlighting the role of ManNAc in the biosynthetic pathway. ManNAc, the only neutral intermediate in the sialic acid biosynthesis pathway, is thought to enter cells via passive diffusion or a plasma membrane transporter, as shown in in vitro systems (Bardor et al., 2005; Hirschberg et al., 1976; Jones et al., 2004). Glycan denotes glycolipids/glycoproteins. Created with BioRender.com. **(B)** Experimental workflow for GSL analysis: (1) extraction and purification, (2) enzymatic digestion, (3) fluorescent labeling, and (4) normal-phase HPLC. A 2-AA-labeled glucose homopolymer ladder was used to assign glucose unit (GU) values, and a 2-AA-labeled BioQuant chitotriose standard was used for glycan quantification. Labeled glycans released from GSLs were digested for 16 hours at 37°C with sialidase A. Following digestion, glycans were separated from the enzyme using Microcon-10 filters and analyzed by HPLC. Charged GSL species (labelled “C” on the x-axis) were identified by the disappearance of corresponding peaks after sialidase A digestion, whereas neutral species (labelled “N” on the x-axis) showed no change or increased intensity. Peaks eluting at GU 3.35 and 4.17 likely correspond to GM2 and GM1a, respectively, which persist post-digestion due to internal sialic acid residues, and are considered charged. Quantification was performed by integrating peak areas relative to the chitotriose standard, with GSL abundance normalized to total protein. **(C)** Lysotracker staining-FACS analysis of HEK-293T cells treated with vehicle (PBS) or 5 mM ManNAc for 20 hours (n=4 replicates per genotype-treatment group). **(D)** Quantification of the ratio of charged to neutral GSL species in cells treated for 20 hours, normalized to the PBS-treated HEK-293T wild-type cells. Each group contains n=5-6 replicates per genotype-treatment. **(E)** As in (D), following a 24-hour treatment. Each group contains n=5-6 replicates per genotype-treatment. Data are presented as mean ± SEM, with statistical significance determined by one-way ANOVA followed by Šídák's multiple comparisons test with *p* -values as indicated.

## Description

Lysosomal free sialic acid storage disorder (FSASD) is an ultra-rare, autosomal recessive neurodegenerative disease resulting from pathogenic variants in *SLC17A5* (Adams and Wasserstein, 2020; Verheijen et al., 1999), which encodes the lysosomal membrane transporter, sialin. Sialin mediates the efflux of sialic acid and other acidic hexoses from lysosomes into the cytosol (Blom et al., 1990; Courville et al., 2010; Morin et al., 2004; Pietrancosta et al., 2012). Loss of functional sialin results in lysosomal accumulation of unconjugated (“free”) sialic acid, primarily N-acetylneuraminic acid (Neu5Ac), the most abundant sialic acid in mammals (Schauer, 2009; Varki, 1992). FSASD comprises three main clinical subtypes: infantile FSASD (MIM#269920), an intermediate severe form, and attenuated FSASD (MIM#604369), also referred to as Salla disease, which is typically associated with homozygosity for the common founder variant, p.Arg39Cys (Adams and Wasserstein, 2020; Huizing et al., 2021). Approximately 250 cases have been identified globally, with an estimated 75% of affected individuals carrying the Finnish founder missense variant *SLC17A5* c.115C>T (p.Arg39Cys) in homozygous or compound heterozygous state (Aula et al., 2000; Barmherzig et al., 2017; Huizing et al., 2021). Genotype-phenotype correlations have been described in several studies (Adams and Wasserstein, 2020; Aula et al., 2000; Kleta et al., 2003; Varho et al., 2000).

To date, cellular models of FSASD have been limited to dermal fibroblasts and peripheral blood leukocytes obtained from affected individuals, which have provided mechanistic insights into pathophysiology of the disorder (Baumkotter et al., 1985; Mancini et al., 1992; Mendla and Cantz, 1984; Pitto et al., 1996). Importantly, studies employing fibroblasts have highlighted disrupted turnover of sialoglycoconjugates, including sialoglycoproteins and gangliosides (i.e., sialylated glycosphingolipids or GSLs) in FSASD cells (Chigorno et al., 1996; Pitto et al., 1996). GSLs, including gangliosides, are vital components of the eukaryotic plasma membrane, playing key roles in cell–cell recognition and interaction, regulation of signal transduction pathways, and modulation of inflammatory responses (Schnaar et al., 2022).

While the primary defect in FSASD, namely lysosomal accumulation of free sialic acid is well established, the downstream extra-lysosomal consequences of disrupted sialic acid efflux and its potential impact on sialic acid availability for glycoconjugate sialylation remain poorly understood. The sialin-mediated salvage pathway is presumed to supplement cytoplasmic sialic acid pools used for activation to CMP-Neu5Ac and subsequent incorporation into nascent glycans. However, the relative contribution of this route to steady-state sialylation, particularly in metabolically active or differentiated cells, has not been rigorously quantified. In this study, we utilized a SLC17A5-deficient HEK-293T cell model, generated via CRISPR-Cas9-genome editing, to: (1) assess the relative abundance of GlcCer-derived gangliosides (charged) and asialo-GSL (neutral) in the presence of sialin deficiency; and (2) investigate whether supplementation with N-acetylmannosamine (ManNAc), a metabolic precursor of sialic acid, can affect GSL sialylation in the absence of salvage. Given that sialin deficiency disrupts the export of free sialic acid from lysosomes, thereby theoretically reducing its cytoplasmic availability for activation to CMP-sialic acid and subsequent incorporation into sialylated glycoconjugates, we hypothesized that exogenous supplementation with ManNAc—an upstream precursor in the *de novo* sialic acid biosynthetic pathway ( **Fig. 1A** )—could enhance cytoplasmic sialic acid levels and thereby improve ganglioside sialylation.

ManNAc has been explored as a therapeutic candidate for GNE myopathy (MIM#605820; NCT04231266), a rare adult-onset neuromuscular disorder (Carrillo et al., 2020). This condition is caused by mutations in the *GNE* gene, which encodes the bifunctional enzyme UDP-N-acetylglucosamine 2-epimerase/ManNAc kinase—the rate-limiting enzyme in sialic acid biosynthesis and a key regulator of cell surface sialylation (Keppler et al., 1999). In GNE mouse models, oral supplementation with ManNAc was shown to improve tissue hyposialylation phenotypes (Malicdan et al., 2009; Niethamer et al., 2012). Supplemental ManNAc, a neutral monosaccharide and the first committed precursor in the sialic acid biosynthetic pathway, can circumvent feedback inhibition by bypassing the rate-limiting enzymatic step catalyzed by UDP-N-acetylglucosamine 2-epimerase ( **Fig. 1A** ).

In this study, we treated HEK-293T wild-type and SLC17A5-deficient cells with either phosphate-buffered saline (PBS) as a vehicle control or 5 mM ManNAc for 20 or 24 hours and subsequently analyzed lysosomal volume using LysoTracker™ staining and GSL abundance/composition before and after sialidase A digestion via normal-phase HPLC ( **Fig. 1B** ). Given that ManNAc supplementation increases sialic acid substrate availability (Peters et al., 2023), it may exacerbate lysosomal storage burden in SLC17A5-deficient cells. Therefore, measuring lysosomal volume via LysoTracker™ staining provides a functional readout to evaluate whether ManNAc treatment results in lysosomal expansion.

In wild-type cells, fluorescently labeled glycans released from GSLs were further digested with sialidase A to distinguish between sialylated (charged) and asialo- (neutral) species ( **Fig. 1B – HPLC callout panel** ). Compared to PBS-treated wild-type cells, PBS-treated SLC17A5-deficient cells displayed a 1.4-fold increase in LysoTracker™ signal intensity ( *p* < 0.0001), consistent with lysosomal expansion, while ManNAc treatment did not significantly alter lysosomal volume in either genotype ( **Fig. 1C** ). GSL profiling revealed a significant reduction in the ratio of charged to neutral species in PBS-treated SLC17A5-deficient cells compared to wild-type controls, with ratios reduced to 0.70 and 0.79 at 20 and 24 hours, respectively ( *p* < 0.0001; **Figs. 1D and 1E)** . ManNAc supplementation increased the charged-to-neutral GSL ratio in wild-type cells by 1.13- and 1.14-fold at 20 and 24 hours, respectively, and partially rescued this ratio in SLC17A5-deficient cells, elevating it from 0.70 to 0.80 at 20 hours and from 0.79 to 0.91 at 24 hours ( *p* < 0.0001; **Figs. 1D and 1E** ). Despite this improvement, the charged-to-neutral GSL ratio in ManNAc-treated SLC17A5-deficient cells remained lower than that observed in wild-type cells treated with either PBS or ManNAc ( **Figs. 1D and 1E** ).

Collectively, our findings suggest that ManNAc supplementation enhances *de novo* sialic acid biosynthesis, as indicated by the increased ratio of charged to neutral GSL species. However, this increase appears insufficient to fully restore GSL sialylation to wild-type levels in the context of sialin deficiency. These results further highlight the intricate interplay between the biosynthetic and salvage pathways of sialic acid metabolism and suggest that sialin-mediated lysosomal export of sialic acid may be necessary for maintaining cytoplasmic sialic acid pools required for GSL sialylation, an essential process with potential downstream consequences for cellular proliferation (Hakomori, 1970; Robbins and Macpherson, 1971), cell cycle regulation (Chatterjee et al., 1975), and differentiation (Varki, 1993). Importantly, our findings demonstrate that alterations in GSL sialylation precede detectable changes in lysosomal volume following ManNAc treatment—at least at 20 hours—suggesting that GSL sialylation may serve as a more sensitive and tractable cellular phenotype for monitoring early responses to this metabolic intervention.

We acknowledge limitations in this study. First, our analyses were limited to two timepoints—20 and 24 hours—following treatment with vehicle or ManNAc. A more comprehensive time course, including earlier timepoints, would allow for finer resolution of the temporal dynamics of ManNAc uptake, metabolic incorporation, and its downstream effects on GSL sialylation. Next, our study was only conducted in HEK-293T cells; further investigation in disease-relevant cell models, such as patient-derived fibroblasts, leukocytes (Sabir et al., 2025b), and iPSC-derived neural cells (Sabir et al., 2025a) as well as FSASD mouse models (Prolo et al., 2009; Sabir et al., 2022; Sabir et al., 2025b; Stroobants et al., 2017) are needed to determine the generalizability of these findings. Moreover, direct quantification of intracellular sialic acid and analysis of glycoprotein sialylation are needed to further investigate the cellular response to ManNAc supplementation. Despite these limitations, this study represents the first investigation of ManNAc as a potential therapeutic compound for disorders linked to sialin deficiency.

## Methods

*Generation and culturing of HEK-293T SLC17A5-deficient cells*

HEK-293T cells were transfected with a plasmid expressing a single guide RNA targeting exon 2 of the *SLC17A5* gene (Harb et al., 2023) and SpCas9 nuclease, followed by nucleofection using the Lonza 4D Nucleofector X-unit. GFP-positive cells were sorted via flow cytometry, isolated, and expanded into monoclonal colonies. Sanger sequencing of the *SLC17A5* exon 2 region identified three clones with biallelic compound heterozygous mutations in *SLC17A5* . Cells were cultured in feeder media containing high-glucose DMEM supplemented with 10% fetal bovine serum, 1% L-glutamine, and 1% penicillin-streptomycin for downstream studies.

*LysoTracker™ staining-FACS analysis*

HEK-293T wild-type and SLC17A5-deficient cells were seeded at a density of 50,000 cells per T-25 flask (n=4 replicates per genotype-treatment group). Cells were incubated overnight at 37°C in 5% CO _2_ . The following day, cells were treated with either phosphate-buffered saline (PBS; vehicle control) or 5 mM N-acetylmannosamine (ManNAc; dissolved in PBS) in feeder media (as described above). After 20 hours of incubation, cells were processed, and relative lysosomal volume was assessed using the fluorescent probe LysoTracker ^TM^ -green DND-26 as previously described (te Vruchte et al., 2014). Briefly, cells were harvested and washed twice with PBS, followed by staining with 200 nM LysoTracker™ for 10 minutes in the dark. After staining, cells were washed again with PBS and resuspended in buffer containing 5 µg/ml propidium iodide to exclude non-viable cells. Samples were immediately analyzed on a BD FACS-Canto II flow cytometer (Becton Dickinson). For each sample, a minimum of 10,000 events were acquired using the DIVA software (Becton Dickinson, version *.0.1), and relative fluorescence intensity was quantified using the FlowJo software (FlowJo, LLC; version 10). A representative flow cytometry plot of LysoTracker™ staining in wild-type and SLC17A5-deficient HEK-293T cells is shown in the extended data (HEK_ManNAc flow cytometry plot).

Note, the concentration of 5 mM ManNAc was selected based on prior studies demonstrating that this dose effectively restores polysialylation to near wild-type levels in cultured HEK-293T wild-type and *GNE* knock-out cells (Neu et al., 2024).

*Profiling cellular GSLs*

HEK-293T wild-type and SLC17A5-deficient cells were plated at a density of 200,000 cells per T-25 flask, with n=5-6 replicates per genotype-treatment group. Cells were treated with either PBS vehicle or 5 mM ManNAc in feeder media (described above). At 20 and 24 hours post-treatment, cells were harvested and lysed in water using three freeze-thaw cycles.

GSL profiling was performed as previously described (Priestman et al., 2024). Briefly, lipids were extracted overnight at 4°C using chloroform:methanol. GSLs were subsequently isolated by solid-phase extraction on C18 columns. Eluted fractions were evaporated under nitrogen at 42 °C, and dried samples were subjected to enzymatic digestion using recombinant ceramide glycanase to release glycans from complex GSLs. Released oligosaccharides were fluorescently labeled with anthranilic acid (2-AA), and excess label was removed using DPA-6S solid-phase extraction columns. Purified 2-AA–labeled glycans were analyzed by normal-phase high-performance liquid chromatography (HPLC). A 2-AA–labeled glucose homopolymer ladder was used to assign glucose unit (GU) values. Glycan quantification was performed by integrating peak areas relative to a 2-AA–labeled BioQuant chitotriose standard. GSL abundance was normalized to total protein content, determined using the bicinchoninic acid (BCA) assay following standard procedures.

*Sialidase A digestion and calculation of charged to neutral GSL species ratio*

Labeled glycans released from HEK-293T GSLs were digested for 16 hours at 37°C in a total volume of 10 µl with sialidase A according to manufacturer’s instructions. Following digestion, labeled glycans were separated from enzyme using Microcon-10 Centrifugal Filters before HPLC as described above.

Charged GSL species were identified by the loss of corresponding peaks following sialidase A digestion, while neutral species exhibited no change or an increase in peak intensity, reflecting the sequential degradation of gangliosides ( **Fig. 1B – HPLC callout panel** ). Notably, peaks eluting at GU 3.35 and 4.17 likely correspond to GM2 and GM1a, respectively ( **Fig. 1B – HPLC callout panel** ). These gangliosides contain internal (non-terminal) sialic acid residues, which are not cleaved by sialidase A, explaining the persistence of their peaks post-digestion. Thus, these two peaks are considered to be charged. The charged-to-neutral GSL species ratio was calculated by summing all charged species and dividing by the sum of neutral species, then expressed as a percentage relative to the HEK-293T wild-type PBS reference group.

*Statistical analyses*

Statistical analyses were conducted using ordinary one-way ANOVA with Šídák's multiple comparisons test using GraphPad Prism (GraphPad, version 10.0.3). A two-tailed α-value for significance was set at 0.05.

## Reagents

**Table:** 

**Reagent**	**Supplier**	**Catalog number**
DMEM-high glucose	Sigma-Aldrich	D5671
Fetal bovine serum	Sigma-Aldrich	F7524
Penicillin-streptomycin	Sigma-Aldrich	P0781
L-Glutamine (200 mM)	ThermoFisher Scientific	25030024
Phosphate-buffered saline (PBS); no calcium, no magnesium	ThermoFisher Scientific	14190250
Trypsin-EDTA (0.25%), phenol red	ThermoFisher Scientific	25200056
N-acetylmannosamine (ManNAc)	New Zealand Pharmaceuticals	Custom order
LysoTracker™ Green DND-26	ThermoFisher Scientific	L7526
Propidium iodide solution	Millipore Sigma	537060
Ceramide glycanase (rEGCase)	GenScript	Custom order
Kinesis SPE Columns: TELOS® C18(EC) 100mg/1ml SPE Columns	Cole-Parmer	EW-06476-81
Discovery® DPA-6S SPE Tube	Millipore Sigma	52624-U
Anthranilic acid (2-AA)	Sigma-Aldrich	A89855
Glucose homopolymer ladder (2-AA labeled)	Ludger Ltd.	CAA-GHP-30
Ludger-BioQuant chitotriose standard (2-AA labeled)	Ludger Ltd.	BQ-CAA-CHI-01
Bicinchoninic acid solution	Millipore Sigma	B9643-1L
AdvanceBio Sialidase A	Agilent	GK80040
Microcon-10 Centrifugal Filters	Millipore Sigma	MRCPRT010

## Data Availability

Description: HEK_ManNAc flow cytometry plot. Resource Type: Image. DOI: https://doi.org/10.22002/vh16y-vxj14
